# Purification of Nanoparticles by Size and Shape

**DOI:** 10.1038/srep27494

**Published:** 2016-06-08

**Authors:** James D. Robertson, Loris Rizzello, Milagros Avila-Olias, Jens Gaitzsch, Claudia Contini, Monika S. Magoń, Stephen A. Renshaw, Giuseppe Battaglia

**Affiliations:** 1 Department of Chemistry, University College London, London, United Kingdom; 2 Department of Biomedical Science, University of Sheffield, Sheffield, United Kingdom; 3 Department of Infection and Immunity, University of Sheffield, Sheffield, United Kingdom; 4 MRC Centre for Developmental and Biomedical Genetics, University of Sheffield, Sheffield, United Kingdom; 5 London Interdisciplinary Biosciences Consortium, Division of Biosciences, University College London, London, United Kingdom; 6 Department of Chemistry, University of Basel, Basel, Switzerland

## Abstract

Producing monodisperse nanoparticles is essential to ensure consistency in biological experiments and to enable a smooth translation into the clinic. Purification of samples into discrete sizes and shapes may not only improve sample quality, but also provide us with the tools to understand which physical properties of nanoparticles are beneficial for a drug delivery vector. In this study, using polymersomes as a model system, we explore four techniques for purifying pre-formed nanoparticles into discrete fractions based on their size, shape or density. We show that these techniques can successfully separate polymersomes into monodisperse fractions.

Size and shape are important parameters for nanoparticle-based drug delivery^1^, that control the kinetics of internalisation^2^, biodistribution^3^, cellular membrane deformability^4^, and cargo loading efficiency^5^. It has been shown that smaller nanoparticles escape natural body clearance mechanisms (*i.e.*, the reticuloendothelial system) more efficiently and, hence, circulate longer in the blood^6^. In addition to size, shape has played an important role in studies demonstrating that cylindrical nanoparticles interact with cells very differently to spherical ones, resulting in dramatic changes in bioavailability^7^. Yet, when it comes to nanoparticle fabrication, often the production of homogenous batches is challenging. The formation of nanoparticles such as micelles or vesicles by self-assembly methods inherently results in higher heterogeneity due to the complex thermo-dynamics and kinetics involved. On the other hand, the heterogeneity of self-assembled systems makes them a good model for studying size and shape separation techniques. Polymersomes, as synthetic vesicles formed through the self-assembly of block copolymer amphiphiles, are a good model for these studies^8^, since a wide range of shapes and sizes of vesicles can be formed in the self-assembly processes. Additionally, these structures are often stable for long periods of time since the chain entanglement and interdigitation of the hydrophobic polymer blocks ensure high mechanical stability^9^,^10^.

A number of techniques have previously been employed to separate nanoparticles by size and shape^11^, and several methods have been developed to purify subpopulations of particles after their assembly. Size exclusion chromatography, for example, allows for the separation of both hard and soft nanoparticles according to their size^12^,^13^,^14^,^15^. Similarly, various mass- and density-based purification methods enable the separation of nanoparticles according to their shape (*e.g.* discoidal from spherical-like nanoparticles)^16^,^17^,^18^. All of these techniques can successfully produce good sub-populations of nanomaterials displaying uniform physicochemical properties. It is worth noting that the assembly procedure may lead to a simultaneous production of nanoparticles with several different properties. In this case, the assembled nanoparticles might contain spherical vesicles together with micelles, worm-like structures, and tubular and genus (*i.e.* ‘donut’- or ‘pretzel’- like) vesicles.

In this work, we aim to explore the analytical approaches for separating different sizes and morphologies of polymersomes pre-assembled from a pH-sensitive block copolymer – poly(2-(methacryloyloxy) ethyl-phosphorylcholine)-co-poly(2-(diisopropylamino)ethyl methacrylate) (PMPC_25_-PDPA_70_). We show that polymersomes can be separated into distinctive sizes, shapes and structures using filtration, centrifugation, size-exclusion chromatography and density gradient centrifugation. We aimed to provide with this work a comprehensive overview of techniques required for purification of heterogeneous nanoparticle solutions into homogenous fractions, which is crucial for future biomedical applications.

## Materials and Methods

### Preparation of PMPC_25_-PDPA_70_ polymersomes

PMPC_25_-PDPA_70_ was prepared by atom-transfer radical polymerisation (ATRP) as previously described^19^. In brief, the initiator was mixed with 25 equivalents of MPC, dissolved in ethanol, degassed and CuBr (1 eq.) and bipyridin (2 eq.) were added. After one hour the polymerisation mixture was assessed by NMR for complete conversion. A degassed solution of 70 eq. DPA dissolved in ethanol was then added and the polymerisation again ran until completion (16 hours). Salts and organic molecules were removed by filtration through silica gel and consecutive dialysis. The final solution was freeze-dried to yield the PMPC_25_-PDPA_70_ polymer. Gel Permeation Chromatography (GPC) was performed on GPC Max (Malvern Instruments) using Novemax Guard and Analytical column (PSS Polymer) in water with 0.25 vol-% Triflouroacetic acid. Figure S1 shows a GPC trace characterisation of the PMPC_25_-PDPA_70_ polymer. Self-assembly of polymersomes was initiated using the thin film rehydration method as described previously^11^, or the pH-switch method. In the latter, a known weight of PMPC_25_-PDPA_70_ polymer is dissolved in acidic Phosphate Buffered Saline (PBS) and the pH is re-adjusted to 2 by the addition of 1 M hydrochloric acid. Using a syringe pump containing 0.5 M sodium hydroxide in a 5 mL syringe the pH is slowly raised at a speed of 10 *μ*L/min under constant stirring with a magnetic stirring bar in the solution until the pH 7.4 is reached.

### Purification by filtration

Spectrum’s KrosFlo Research II*i* System and filter modules were purchased from Spectrum labs and were used following manufacturers instructions. Briefly, 10 mL of polymersomes at a polymer concentration of 10 mg/mL were diluted to 50 mL with PBS. The dilute polymersome solution was aliquoted into polystyrene sample tubes and attached to the KrosFlo research system with a 50 nm hollow fibre filter module. The filtration was started with the flow rate of 2 mL/minute. After the retained volume was reduced to 2 mL it was re-diluted to 50 mL and the process was repeated. To concentrate polymersome samples, a hollow fibre module with pores of 10 kDa was utilised.

### Purification by centrifugation

The initial step of polymersome purification by size involved removal of micelles from the solution using the KrosFlow filtration system. The polymersomes were centrifuged at 500 Rotational Centrifugal Force (RCF) for 20 minutes. The resulting pellet was removed and resuspended in PBS. This fraction contained the largest aggregate fraction. The supernatant was then re-centrifuged at 2000 RCF for 20 minutes and the pellet was removed and re-suspended, constituting fraction 1. This was repeated with further 20-minute centrifugations at 5000, 10000, 15000 and 20000 RCF.

### Purification by GPC

For separation of polymersomes by GPC, micelles and aggregates were removed as described above and the remaining polymersome solution was concentrated to approximately 200 μL using a 500 kDa MicroKros filter module. The solution was then placed in a glass liquid chromatography column containing Sepharose 4B. The fractions were collected in a 96-well plate. Dynamic Light Scattering (DLS) measurements were performed on a Zetasizer Nano ZS (Malvern Ltd.) as described previously^12^.

### Purification by density gradient centrifugation

Density gradient centrifugation was performed using solutions of 5, 10, 15, 20, and 25% w/v sucrose dissolved in PBS. Aliquots of 200 μL of each solution were carefully layered one on the top of each other in the order from the densest to the least dense within a 1.5 mL microcentrifuge tube, while avoiding mixing between the layers. Finally, 150 μL of the solution containing a mixture of rhodamine-labelled vesicles of different shapes (spheres and tubes) prepared by film rehydration was deposited at the top of the sucrose layers and the microcentrifuge tube was centrifuged at 20000 RCF for 2 hours. After centrifugation, 20 μL of solution from each layer was collected and analysed by Transmission Electron Microscopy (TEM).

### Polymersome characterisation

TEM analysis was performed using a FEI Tecnai G2 Spirit electron microscope and/or a JEOL 2100 operating at 200 kV equipped with a CCD camera Orius SC2001 from Gatan. Copper grids were glow discharged and the sample was adsorbed onto the grid. The sample was then stained with 0.75 wt% phosphotungstic acid (PTA) adjusted to pH 7.4 with NaOH. All the TEM analyses were carried out with dried samples.

DLS analyses were carried out using a Zetasizer Nano ZS (Malvern Ltd.) at a copolymer concentration of 0.25 mg/mL. DLS measurements were based on 12−14 runs, 10-second sub-runs. Samples were analysed at 25 °C with a scattering angle of 173° and a 633 nm HeNe laser based on a material refractive index (RI) of 1.59, a dispersant refractive index of 1.330 and a viscosity of 0.89.

## Results and Discussion

### Why use polymersomes as a model for nanoparticle purification?

In this study, we have chosen polymeric vesicles, polymersomes, as a nanoparticle model for demonstrating the efficiency of different purification techniques. We have previously shown that the average polymersome size is influenced by the preparation technique employed, but each method produces a wide distribution of sizes around their mean diameters^20^. The most energetically favourable geometry for a polymersome (and any other vesicle) is a sphere. However, the process of membrane wrapping can only occur above a critical radius typically controlled by the size of the single building block^21^. Above this critical radius, the energetic penalty required for an amphiphilic membrane to wrap into a vesicle is not dependent on the final diameter^19^. This means the self-assembly of spherical polymersomes is not associated with a strong size selective bias. The method of aggregate formation can also influence the assembled nanoparticle shape and structure^22^. The final morphology of nanoparticles depends on the distribution of molecules across the two monolayers. Membrane wrapping can result in different non-spherical shapes that are stabilised by the high molecular weight of block copolymers^23^. One way to improve homogeneity of vesicle size and shape is to provide additional energy to break and re-form the vesicles several times. For example, phospholipid-based vesicles, liposomes, are commonly homogenised by a combination of sonication and extrusion in order to break up larger structures and reduce the size diversity. However, these techniques are often infeasible for more mechanically robust nanoparticles such as polymersomes. The high molecular weight of polymers used in polymersome preparations ensures the mechanical resistance and therefore breaking of the polymersome membranes would require involvement of substantial forces^24^. Here we use pH-sensitive block copolymers PMPC-PDPA to form polymersomes using two methods: (*i*) solvent switch, exploiting the PDPA sensitivity to pH, and (*ii*) film rehydration.

The former method of self-assembly, referred to as pH-switch, is a version of a solvent switch method, where the solvent’s pH is altered. It is a simple and quick procedure that facilitates the formation of polymersomes in less than an hour. The polymersomes are produced by dissolving polymeric unimers, and by subsequently changing the solubility conditions to drive self-assembly. Under aqueous conditions, with a pH above the polymer pKa, the unimers come together to form structures that shield their hydrophobic blocks from water. As we observed previously, this initial stage is relatively fast and initially leads to the formation of spherical micelles (monolayered polymeric aggregates without an aqueous interior) regardless of the copolymer hydrophobic/hydrophilic ratio^12^. For membrane-forming copolymers the transition from micelles to vesicles (double layered membranes enclosing an aqueous interior) occurs through the formation of different intermediate phases mostly *via* a growth mechanism^25^. However, the critical condition for such a growth is that there is an equilibrium between unimers and assemblies. When the pool of unimers in solution is exhausted, the self-assembly process arrests often leading to the formation of metastable structures such as micelles. Conversely, if a vesicle forms and there is still a pool of unimers in solution, the vesicle starts growing with a misbalance between the inner and the outer layer leading to the formation of high genus vesicles^22^.

For film rehydration methods, the formation of polymersomes occurs via the hydration of a pre-formed copolymer film. During the hydration process, the copolymer forms different structures depending on the copolymer/water ratio^10^. This process is kinetically complex and often leads to the formation of different metastable phases such as multilamellar aggregates^26^, as well as tubular polymersomes^27^.

This broad variety of structures make polymersomes an ideal platform to develop new methods of separation as their properties are highly dependent on their size and shape.

### Cross flow-filtration

**Figure 1:**
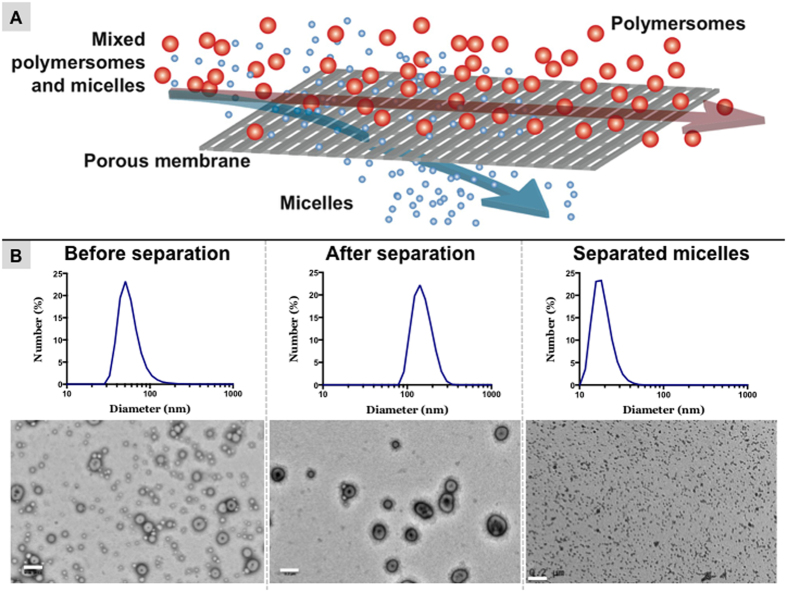
Purification of polymersomes from micelles using the KrosFlo Research Ili System. (**A**) A diagram displaying the method of micelles removal by filtration through 50 nm pores. **(B)** DLS frequency distributions and TEM micrographs of the polymersomes solution before separation (left images), after separation (central images) and the separated micelle solution (right images). Scale bar = 200 nm.

One method of purifying polymersomes based on size is filtration. Dead-end filtration is generally unsuccessful because nanoparticles concentrate at the pores resulting in a “filter cake” that blocks the filter. Cross flow-filtration (CFF) overcomes this problem by providing a flow of particles at a tangent to the pores under high pressure. The pressure allows particles smaller than the pore size to permeate the membrane while the tangential flow prevents the formation of a filter cake (see the representative scheme in Fig. 1A)^28^. After producing polymersomes by the pH-switch method, we used a CFF equipped with membranes with a pore size of 50 nm in diameter to efficiently purify spherical and genus polymersomes from micelles, as shown by DLS and TEM in Fig. 1B. However, the same approach evaluated with DLS and TEM was not applicable to the separation of polymersomes (vesicles) into distinct sizes and shapes. This could be due to the intrinsic soft nature of polymersomes, which can deform in the proximity of the pores if a specific threshold pressure is applied. Although DLS provides a rapid measurement of a sample size distribution, it is important to note that DLS results can be less accurate in samples with polydisperse or agglomerated nanoparticles. Similarly, a single TEM image may not be completely representative of the entire sample. Therefore, it is good practice to include both measurements when assessing particle size. In general, DLS data presented here were in agreement with TEM images. Polymersomes purified from micelles with filtration using the KrosFlo Research IIi System were observed as a polydisperse mixture of sizes in the TEM image, and similarly the DLS reported a relatively high polydispersity index (PDI) of 0.283.

### Differential centrifugation

**Figure 2:**
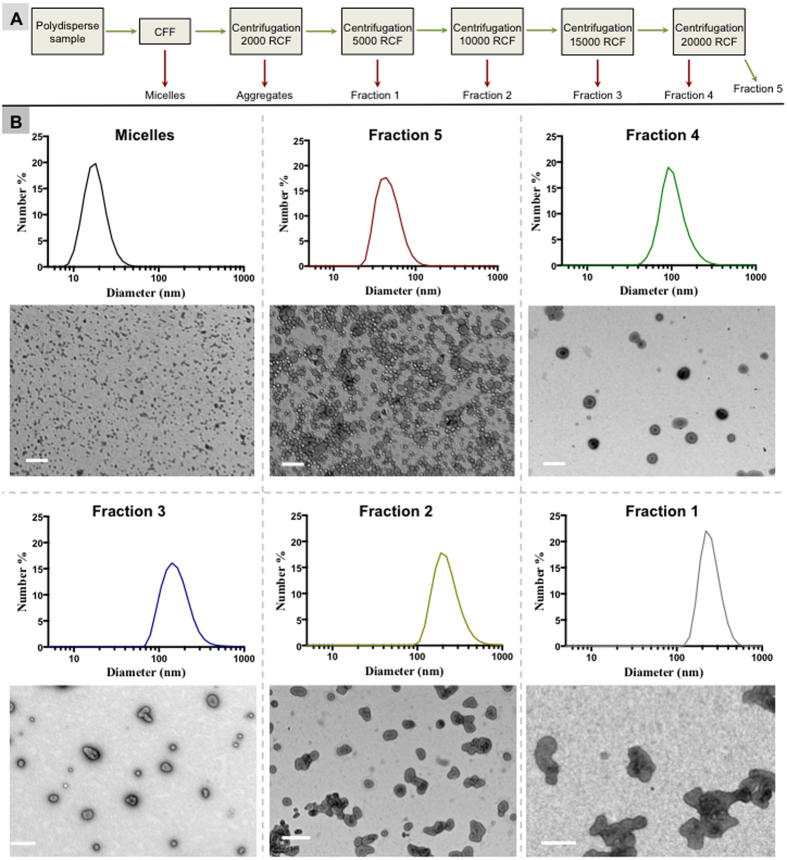
Purification of polymersomes with DC. (**A**) Cartoon explaining the separation protocol. Micelles are first removed using the KrosFlo hollow fiber system. The sample is then centrifuged at 500 RCF and the pellet is resuspended in PBS. The supernatants were then centrifuged consecutively at 2000 RCF, 5000 RCF, 10000 RCF, 15000 RCF, and 20000 RCF. After each centrifugation, the pellet was separated and resuspended. **(B)** DLS size distributions and the corresponding TEM micrographs displaying the separated fractions in ascending size order. Scale bar = 200 nm.

Centrifugation is one of the most commonly used techniques in bioscience research, mostly for separation of mixtures by size and density. Differential centrifugation (DC) is used to separate multiple fractions within a sample. In this method, the mixture is centrifuged multiple times, and after each run the pellet is removed and the supernatant is centrifuged at a higher centrifugal force (Scheme in Fig. 2A).

In our experimental approach, polymersomes were formed by the pH-switch method. Figure S2 in supporting information shows a TEM picture of these polymersomes before carrying out any further purification steps. It is evident that the resulting sample was highly polydisperse in terms of both size and shape, and contained a large population of micellar structures. Micelles have been thus preliminary removed using the previously described CFF system. The remaining polymersomes were then separated into different size fractions by DC. TEM micrographs for each fraction after the DC, and their respective DLS analyses, can be seen in Fig. 2B. This method was quick, simple and resulted in a successful separation of the polydisperse sample into distinct, more homogenous size fractions without any loss of material. However, further experiments showed that DC method cannot be used to separate different shapes of polymersomes as the high-genus structures were observed in multiple fractions. Recently, we have observed that the low RCF fractions of polymersomes formed by film rehydration and purified by DC are enriched in tubular polymersomes^29^. However, the separation was not complete and, therefore, the level of purification was not satisfying. For this reason, we focused then on size exclusion chromatography.

### Size exclusion chromatography

**Figure 3:**
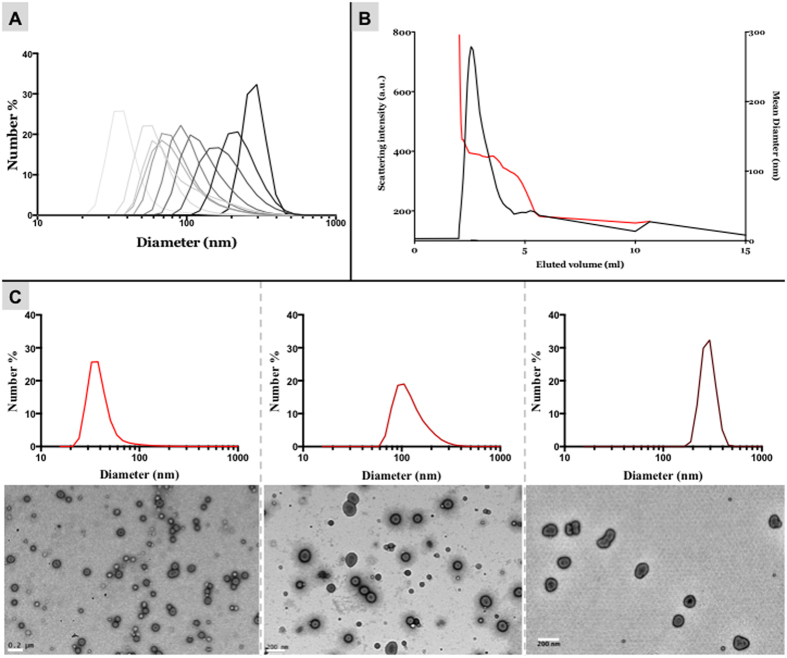
Size separation using two columns. **(A)** Representative DLS frequency distribution of the different fractions separated by two in series SEC columns. **(B)** Chromatogram showing the elution of polymersomes from the SEC column as a function of scattering intensity (black curve) and relative average size (red curve). **(C)** DLS frequency distributions and TEM micrographs for three representative fractions. Scale bars = 200 nm.

The next separation technique explored was size exclusion chromatography (SEC), which separates particles based on their hydrodynamic volume. As a mixture moves through the stationary phase of a SEC column, typically made of packed hydrophilic polymeric beads, smaller molecules meander in and out of pores within the gel, whereas larger molecules cannot penetrate into the pores, allowing them to pass through the stationary phase quicker and elute from the column earlier. SEC is commonly used to separate free small molecules from those encapsulated within nanoparticles, however, the use of the method for the separation of distinct sizes and shapes of nanoparticles can be more challenging^30^. To improve the resolution of SEC, the polymersome solution was concentrated into a small volume using the KrosFlo Research II*i* System with a 10 kDa pore size membrane. This reduced the time required for the liquid to be absorbed by the stationary phase. The eluted liquid was passed twice into the same column, doubling the effective column length. The eluted sample was then collected into a 96-well plate and the separated fractions’ mean diameter were calculated from the DLS measurements. The data is reported using each sample scattering intensity (indicative of the polymersomes size and concentration) and the mean size (diameter) as a function of the elution volume. The relative frequency size distribution of each fraction of polymersomes is plotted in Fig. 3A. The resulting chromatogram is shown in Fig. 3B that suggests the effective separation of the polymersomes into several sizes. This technique was effective at separating the sample into many discrete monodisperse size fractions. Examples of three fractions separated by this method, and analysed by TEM, are shown in Fig. 3C. The monodispersity of the fractions separated by SEC was improved as compared to the samples purified by the DC technique. However, this method is more time consuming and may result in a material loss within the SEC column.

We thus came to the preliminary conclusion that CFF can be an ideal tool for separating micellar structures from vesicles. At the same time, both DC and SEC were found to be optimal for purifying polymersomes according to their size, with their own advantages and disadvantages. However, none of these techniques allowed us to carry out a complete separation by nanoparticle shape. Each samples still contained high level of high genus structures (in the case of polymersomes preparation via pH-switch method), or tubular vesicles (in case of a film rehydration method preparation). We thus explored another technique, namely density gradient centrifugation (DGC), a method widely employed in biochemistry and molecular biology to successfully separate sub-cellular fractions and organelles, according to their density.

### Density gradient centrifugation

**Figure 4:**
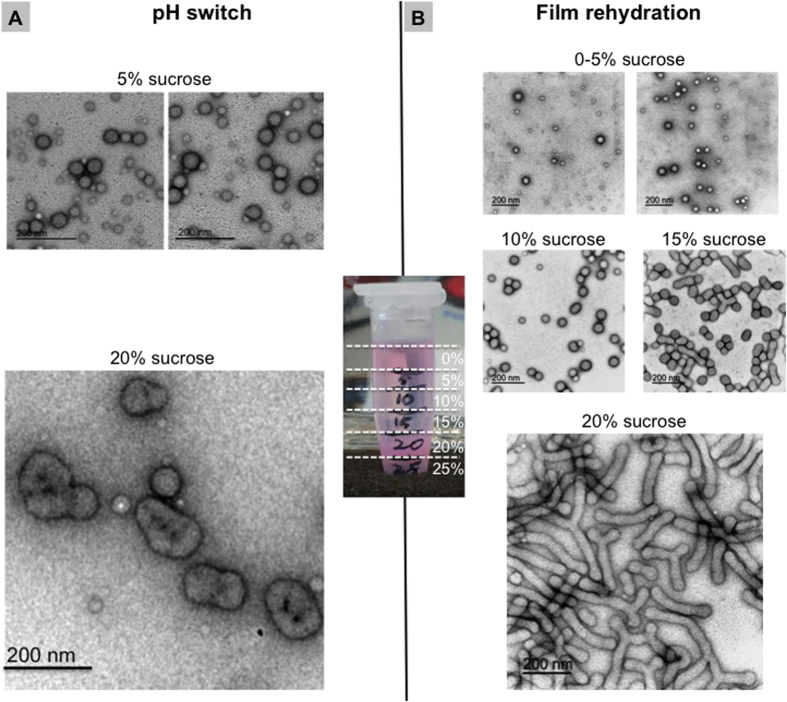
Separation of polymersomes using density gradient centrifugation with a discontinuous gradient of sucrose. (Centre of the picture) A photograph showing the result of a density gradient centrifugation. The rhodamine-labelled polymersomes are first loaded on the top layer. The centrifugation will result in the formation of two major fractions at 5% w/v and 20% w/v of sucrose. (**A**) TEM analyses of polymersomes formed by pH switch, which were recovered from the 5% w/v layer (top), and 20% w/v layer (bottom). (**B**) TEM investigations of polymersomes formed by film rehydration and recovered from 4 layers of sucrose: 5% w/v (top), 10% w/v and 15% w/v (middle), and 20% w/v (bottom). Scale bars = 200 nm.

Finally, we tested DGC as a technique for purification of polymersomes according to their shape. Sucrose DGC allows a mixture to be separated into different fractions based on the particle density. Non-spherical polymersomes tend to have a larger density than spherical ones as the relative internal aqueous volume is smaller and hence they posses a larger polymer/water ratio. To test this, a sample of polymersomes formed by film rehydration was placed on a preformed discontinuous sucrose density gradient by layering successively lower sucrose densities into a 1.5 mL microcentrifuge tube. The layers contained 0% (*i.e.*, the polymersomes solution in PBS), 5%, 10%, 15%, 20% and 25% w/v sucrose in PBS. The sample was then centrifuged for 2 hours at 20000 RCF yielding nanoparticles of different densities suspended in each layer. Polymersomes formed by pH-switch were recovered from the 5% w/v, and 20% w/v sucrose layers (all other fractions did not contain nanoparticles). The 5% w/v sucrose fractions, displayed in Fig. 4A (top), contained a mixture of spherical polymersomes with different sizes. Particles recovered from the 20% w/v sucrose layer contained predominantly large high genus structures (Fig. 4A, bottom). An additional electron micrograph of the 20% w/v sucrose fraction is shown in the Supplementary Information (Fig. S3). The shape and the fold of genus (1-fold, double fold, triple fold, etc.) of the nanoparticles in the 20% w/v sucrose layer varied. The differential density between the two populations can be easily explained by comparing the ability to encapsulate water with the spheres having larger volumes than the high-genus structure. This means that a 100 nm sphere has half the number of copolymer chains compared with a 100 nm torus (*i.e.* 1-fold genus) with a 10 nm hole. Thus, the particle density of spherical polymersomes is always lower than tubular and genus polymersomes.

In a similar way to the pH-switch, the film rehydration technique can result in the formation of a mixed vesicle population of different shapes. However, film rehydration of a polymeric film results in the formation of tubular vesicles instead of genus structures, in addition to spherical vesicles. The tubular vesicles are transitional structures that form during rehydration of the polymer film, and eventually “pearl” into smaller tubes and then further into spherical polymersomes^11^. The higher particle density of tubular polymersome membranes should enable these structures to be purified from spherical polymersomes using DGC. Therefore, this possibility was explored using DGC methodology as described earlier for the separation of genus and spherical particles, however, this time, nanoparticles were prepared using the film rehydration method. The post-centrifugation density gradient for rhodamine-conjugated PMPC-PDPA polymersomes prepared by film rehydration is shown in Fig. 4B. The method yielded four separate fractions. The fraction at 0% to 5% w/v sucrose concentration (Fig. 4B, top) contained the smallest spherical polymersome dispersion, the 10% w/v sucrose fraction (Fig. 4B, middle) contained monodisperse spherical polymersomes, the 15% w/v sucrose fraction (Fig. 4B, middle) contained a mixture of spherical and short elongated polymersomes, and finally the 20% w/v sucrose fraction contained long branched and un-branched tubular polymersomes. In order to quantify the size distribution of the individual fractions isolated using DGC, the TEM images were analysed using ImageJ software. In particular, the collected 0% fraction contained polymersomes having an average diameter range of 10–20 nm. The vesicles present in the 5% w/v fraction were 20–40 nm in diameter, those in the 10% w/v fraction were 40–60 nm, while in the 15% w/v sucrose band the polymersomes were 60–80 nm, although there was also a significant number of vesicles in the diameter range of 90 to 150 nm (see Fig. S4 in supporting information). The broad size distribution of the 20% w/v fraction is due to the presence of tubular vesicles (Fig. S4). The last graph of Fig. S4 displays an overview of all the analysed samples. The DGC has proven to be a very effective method that allowed to purify the film rehydrated nanoparticles into the distinct shape fractions. As shown in Supplementary Information (Fig. S4), these nanoparticles were initially a highly polydisperse mixture of tubular and spherical polymersomes with varying diameters. The DGC provided an easy and effective way of separating these structures with minimal loss of sample during the process, and enabled the sample to be concentrated at the interface between two different sucrose bands. This is because the particles get trapped at the density equilibrium.

## Conclusions

Purifying nanoparticles into monodisperse fractions is essential to understand the physical properties of nanoparticles, such as size and shape, as well as to minimise the variability in biological applications. For purification of nanoparticles by size, other groups have explored methods such as magnetic field flow fractionation filtration^31^, size exclusion chromatography^32^, size selective precipitation^33^, density gradient centrifugation^34^, and cross-flow filtration^35^. Similarly, methods for purifying nanoparticles by shape include centrifugation^36^, gel electrophoresis^37^, and density gradient centrifugation^38^. However, these reports have predominantly focused on “hard” nanoparticles such as gold nanoparticles and carbon nanotubes. Moreover, many of these methods cannot be applied to “soft” nanoparticles of tens to hundreds of nanometres in diameter.

The interesting physico-chemical and mechanical properties of polymersomes have attracted several application-focused studies. However, the unique polymersome stability, mechanical robustness and the dynamic self-assembly processes are also the major obstacles in the preparation of homogenous samples. Formation of polymersomes by self-assembly inherently leads to the relatively broad size distribution and high variability in the morphology. Non-vesicular structures, such as micelles, as well as tubes and genus, form alongside spherical vesicles. With the aim of consistency and reproducibility in biological and medical applications, it is of paramount importance to introduce new approaches enabling the production of homogenous polymersome preparations. Here, we have compared four different, yet complementary, techniques to separate polymersomes according to their size and shape. CFF, DC and SEC provided a good solution for the size-dependent separation of particles with each technique bearing different advantages and disadvantages. The CFF method is efficient at separating micelles from polymersomes possibly due to a combination of size exclusion and differential fluid dynamics. Although it cannot be used to separate sub-populations of polymersomes according to their size, the CFF method does provide a means of concentrating a polymersome sample, and holds promises in producing consistent scaling up protocols. The DC and SEC-based methods enabled separation of polymersomes of different sizes into distinctive sub-populations of less than 10 nm difference in diameter. However, these purification methods result in a loss in sample concentration. While neither CFF, DC nor SEC allowed for shape-based separation, DGC achieved this goal. We have observed that the different shape of polymersomes correspond to changes in the density of membrane packing, providing the means for their separation by the DGC-based method.

In this study, block copolymer polymersomes have been used as a nanoparticle model for investigations of various purification techniques with the aim to efficiently separate particles by size and shape. Depending on the type of nanoparticles employed, the ideal purification protocol may combine two or more of these techniques. Once nanoparticle solutions are purified, each fraction can be quantified. Therefore, these techniques not only allow samples to be purified to extract the nanoparticles with the desired physical properties, but may also allow different nanoparticle assembly techniques to be assessed in order to determine which techniques produce nanoparticles with the desired physical properties.

Exploring various separation methods, we have demonstrated it is possible to obtain a homogenous polymersome preparation, both in terms of particle size and shape. This work could provide the basis for more consistent results in biological experiments and improve application development in medicine and drug delivery.

## Additional Information

**How to cite this article**: Robertson, J. D. *et al.* Purification of Nanoparticles by Size and Shape. *Sci. Rep.* **6**, 27494; doi: 10.1038/srep27494 (2016).

## Supplementary Material

Supplementary Information

## References

[b1] Robertson J. D., Patikarnmonthon N., Joseph A. S. & Battaglia G. Block Copolymer Micelles and Vesicles for Drug Delivery in Engineering Polymer Systems for Improved Drug Delivery (Ed. Bader R. A. & Putnam A.) 163–188 (Wiley 2014).

[b2] Chithrani B. D., Ghazani A. A. & Chan W. C. W. Determining the size and shape dependence of gold nanoparticle uptake into mammalian cells. Nano Letters 6, 662–668 (2006). 16608261 10.1021/nl052396o

[b3] Owens D. E. & Peppas N. A. Opsonization, biodistribution, and pharmacokinetics of polymeric nanoparticles. International Journal of Pharmaceutics 307, 93–102 (2006). 16303268 10.1016/j.ijpharm.2005.10.010

[b4] Pegoraro C. *et al.* Translocation of flexible polymersomes across pores at the nanoscale. Biomaterials Science 2, 680–692 (2014). 26828800 10.1039/c3bm60294j

[b5] Hurst S. J., Lytton-Jean A. K. R. & Mirkin C. A. Maximizing DNA loading on a range of gold nanoparticle sizes. Analytical Chemistry 78, 8313–8318 (2006). 17165821 10.1021/ac0613582 PMC2525614

[b6] Avila-Olias M., Pegoraro C., Battaglia G. & Canton I. Inspired by nature: fundamentals in nanotechnology design to overcome biological barriers. Ther Deliv 4, 27–43 (2013). 23323779 10.4155/tde.12.126

[b7] Yoo J.-W. & Mitragotri S. Polymer particles that switch shape in response to a stimulus. Proceedings of the National Academy of Sciences 107, 11205–11210 (2010). 10.1073/pnas.1000346107 PMC2895097 20547873

[b8] Messager L., Gaitzsch J., Chierico L. & Battaglia G. Novel aspects of encapsulation and delivery using polymersomes. Current opinion in pharmacology 18, 104–111 (2014). 25306248 10.1016/j.coph.2014.09.017

[b9] Battaglia G. & Ryan A. J. Bilayers and Interdigitation in Block Copolymer Vesicles. Journal of the American Chemical Society 127, 8757–8764 (2005). 15954782 10.1021/ja050742y

[b10] Battaglia G. & Ryan A. J. The evolution of vesicles from bulk lamellar gels. Nat Mater 4, 869–876 (2005). 16379080 10.1038/nmat1501

[b11] Bleul R., Thiermann R. & Maskos M. Techniques To Control Polymersome Size. Macromolecules 48, 7396–7409 (2015).

[b12] Wei G.-T., Liu F.-K. & Wang C. R. C. Shape Separation of Nanometer Gold Particles by Size-Exclusion Chromatography. Analytical Chemistry 71, 2085–2091 (1999). 21662743 10.1021/ac990044u

[b13] Satzer P., Wellhoefer M. & Jungbauer A. Continuous separation of protein loaded nanoparticles by simulated moving bed chromatography. Journal of Chromatography A 1349, 44–49 (2014). 24866563 10.1016/j.chroma.2014.04.093 PMC4048465

[b14] Krieg E., Weissman H., Shirman E., Shimoni E. & Rybtchinski B. A recyclable supramolecular membrane for size-selective separation of nanoparticles. Nat Nano 6, 141–146 (2011). 10.1038/nnano.2010.274 21258332

[b15] Ruysschaert T. *et al.* Liposome retention in size exclusion chromatography. BMC Biotechnology 5, 1–13 (2005). 15885140 10.1186/1472-6750-5-11 PMC1142305

[b16] Johansson E., Lundquist A., Zuo S. & Edwards K. Nanosized bilayer disks: Attractive model membranes for drug partition studies. Biochimica et Biophysica Acta (BBA) – Biomembranes 1768, 1518–1525 (2007). 17451640 10.1016/j.bbamem.2007.03.006

[b17] Iden D. L. & Allen T. M. *In vitro* and *in vivo* comparison of immunoliposomes made by conventional coupling techniques with those made by a new post-insertion approach. Biochimica et Biophysica Acta (BBA) – Biomembranes 1513, 207–216 (2001). 11470092 10.1016/s0005-2736(01)00357-1

[b18] Sánchez-López V., Fernández-Romero J. M. & Gómez-Hens A. Evaluation of liposome populations using a sucrose density gradient centrifugation approach coupled to a continuous flow system. Analytica Chimica Acta 645, 79–85 (2009). 19481634 10.1016/j.aca.2009.04.045

[b19] Du J. Z., Tang Y. Q., Lewis A. L. & Armes S. P. pH-sensitive vesicles based on a biocompatible zwitterionic diblock copolymer. Journal of the American Chemical Society 127, 17982–17983 (2005). 16366531 10.1021/ja056514l

[b20] Battaglia G. & Ryan A. J. Pathways of Polymeric Vesicle Formation. The Journal of Physical Chemistry B 110, 10272–10279 (2006). 16722729 10.1021/jp060728n

[b21] Lasic D. D. The mechanism of vesicle formation. The Biochemical journal 256, 1–11 (1988). 3066342 10.1042/bj2560001 PMC1135360

[b22] Pearson R. T., Warren N. J., Lewis A. L., Armes S. P. & Battaglia G. Effect of pH and Temperature on PMPC-PDPA Copolymer Self-Assembly. Macromolecules 46, 1400–1407 (2013).

[b23] Battaglia G. & Ryan A. J. Neuron-like tubular membranes made of diblock copolymer amphiphiles. Angewandte Chemie-International Edition 45, 2052–2056 (2006). 10.1002/anie.200503334 16493714

[b24] Discher B. M. *et al.* Polymersomes: Tough Vesicles Made from Diblock Copolymers. Science 284, 1143–1146 (1999). 10325219 10.1126/science.284.5417.1143

[b25] Blanazs A., Madsen J., Battaglia G., Ryan A. J. & Armes S. P. Mechanistic Insights for Block Copolymer Morphologies: How Do Worms Form Vesicles? Journal of the American Chemical Society 133, 16581–16587 (2011). 21846152 10.1021/ja206301a

[b26] Battaglia G. & Ryan A. J. Pathways of polymeric vesicle formation. Journal of Physical Chemistry B 110, 10272–10279 (2006). 10.1021/jp060728n 16722729

[b27] Battaglia G. & Ryan A. J. Neuron-Like Tubular Membranes Made of Diblock Copolymer Amphiphiles. Angewandte Chemie 118, 2106–2110 (2006). 10.1002/anie.200503334 16493714

[b28] Rinzler A. G. *et al.* Large-scale purification of single-wall carbon nanotubes: process, product, and characterization. Appl Phys a-Mater 67, 29–37 (1998).

[b29] Robertson J. D. *et al.* pH-Sensitive Tubular Polymersomes: Formation and Applications in Cellular Delivery. ACS Nano 8, 4650–4661 (2014). 24724711 10.1021/nn5004088

[b30] Battaglia G., Ryan A. J. & Tomas S. Polymeric Vesicle Permeability: A Facile Chemical Assay. Langmuir 22, 4910–4913 (2006). 16700572 10.1021/la060354p

[b31] Latham A. H., Freitas R. S., Schiffer P. & Williams M. E. Capillary magnetic field flow Fractionation and analysis of magnetic nanoparticles. Analytical Chemistry 77, 5055–5062 (2005). 16053322 10.1021/ac050611f

[b32] Duesberg G. S., Burghard M., Muster J., Philipp G. & Roth S. Separation of carbon nanotubes by size exclusion chromatography. Chemical Communications, 3, 435–436 (1998).

[b33] Lee J.-S., Stoeva S. I. & Mirkin C. A. DNA-induced size-selective separation of mixtures of gold nanoparticles. Journal of the American Chemical Society 128, 8899–8903 (2006). 16819885 10.1021/ja061651j

[b34] Sun X. *et al.* Separation of Nanoparticles in a Density Gradient: FeCo@C and Gold Nanocrystals. Angewandte Chemie-International Edition 48, 939–942 (2009). 10.1002/anie.200805047 PMC2656675 19107884

[b35] Sweeney S. F., Woehrle G. H. & Hutchison J. E. Rapid purification and size separation of gold nanoparticles via diafiltration. Journal of the American Chemical Society 128, 3190–3197 (2006). 16522099 10.1021/ja0558241

[b36] Sharma V., Park K. & Srinivasarao M. Shape separation of gold nanorods using centrifugation. Proceedings of the National Academy of Sciences of the United States of America 106, 4981–4985 (2009). 19255445 10.1073/pnas.0800599106 PMC2664026

[b37] Hanauer M., Pierrat S., Zins I., Lotz A. & Sonnichsen C. Separation of nanoparticles by gel electrophoresis according to size-and shape. Nano Letters 7, 2881–2885 (2007). 17718532 10.1021/nl071615y

[b38] Xiong B. *et al.* Separation of nanorods by density gradient centrifugation. Journal of Chromatography A 1218, 3823–3829 (2011). 21571285 10.1016/j.chroma.2011.04.038

