# Supplementary material for: Purification of Nanoparticles by Size and Shape
Source: Sci Rep. 2016 Jun 8;6:27494. doi: 10.1038/srep27494 (PMC4897710; doi:10.1038/srep27494)
Supplement: Supplementary Information [file srep27494-s1.doc]

**Purification of Nanoparticles by Size and Shape**

James D. Robertson**^#^** ^1,2,3,4^, Loris Rizzello**^# *^** ^1^, Milagros Avila-Olias^1,2^, Jens Gaitzsch^1^, Claudia Contini^1^, Monika S. Magoń^1,5^, Stephen A. Renshaw^1,2^, Giuseppe Battaglia **^*^** ^1^

^1^*Department of Chemistry, University College London, London, United Kingdom*

^2^*Department of Biomedical Science,* ^3^*Department of Infection and Immunity,* ^4^*MRC Centre for Developmental and Biomedical Genetics, University of Sheffield, Sheffield, United Kingdom, ^5^London Interdisciplinary Biosciences Consortium, Division of Biosciences, University College London, London, United Kingdom*

**SUPPLEMENTARY MATERIAL**


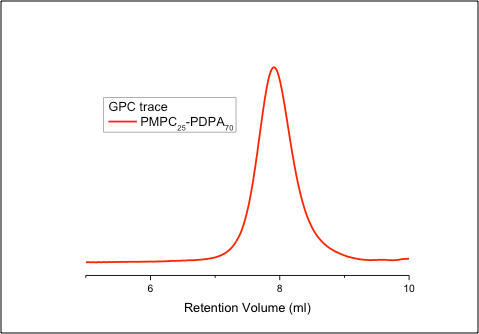


**Figure S1. GPC trace of the PMPC_25_-PDPA_70_ used for our studies.** The Dispersity is 1.24.


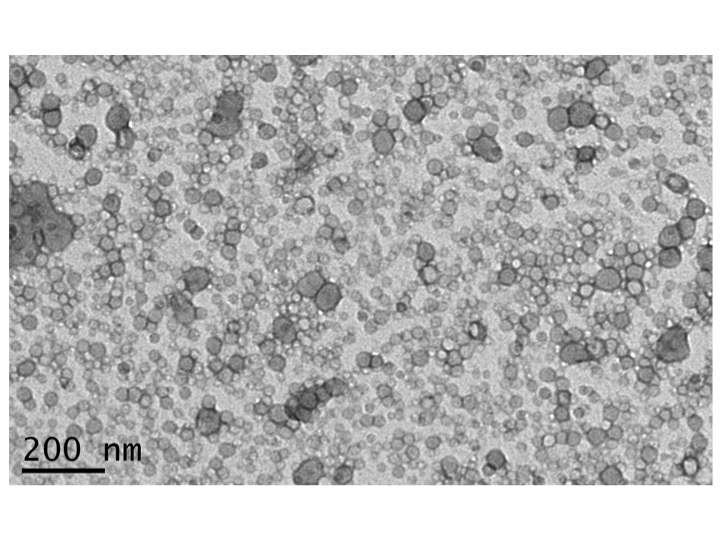


**Figure S2. A TEM photomicrograph of the disomogeneus polymersomes mixture before a differential centrifugation based separation.**


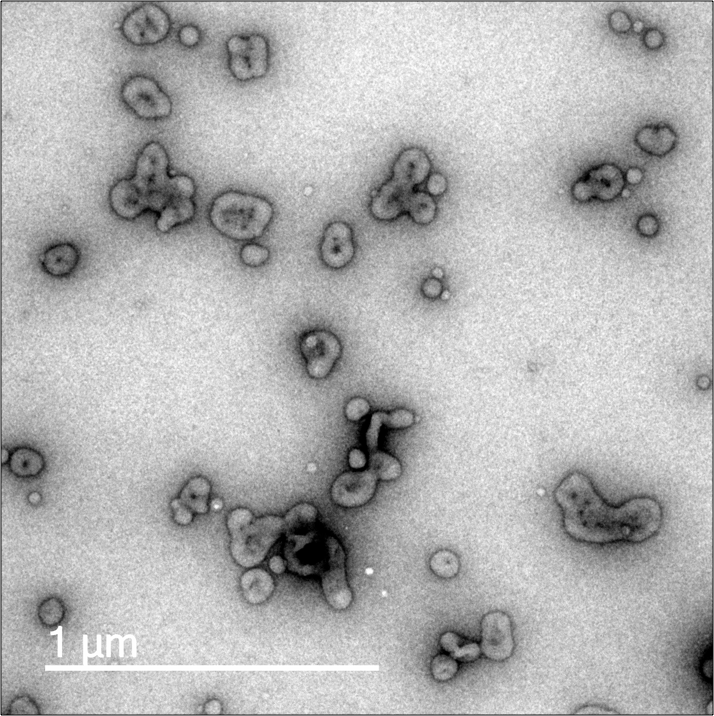
**Figure S3. High genus structures produced by pH switch and purified using density gradient purification.** A TEM photomicrograph of high genus nanoparticles extracted by density gradient centrifugation from the 20% sucrose layer.

**Figure S4. Size distribution graph after DGC.** Polymersomes were prepared by film hydration method and separated through DGC. Each graph corresponds to the samples collected from the 0%, 5%, 10%, 15%, and 20% of sucrose band. ImageJ was used to analyse the nanoparticles size distribution.


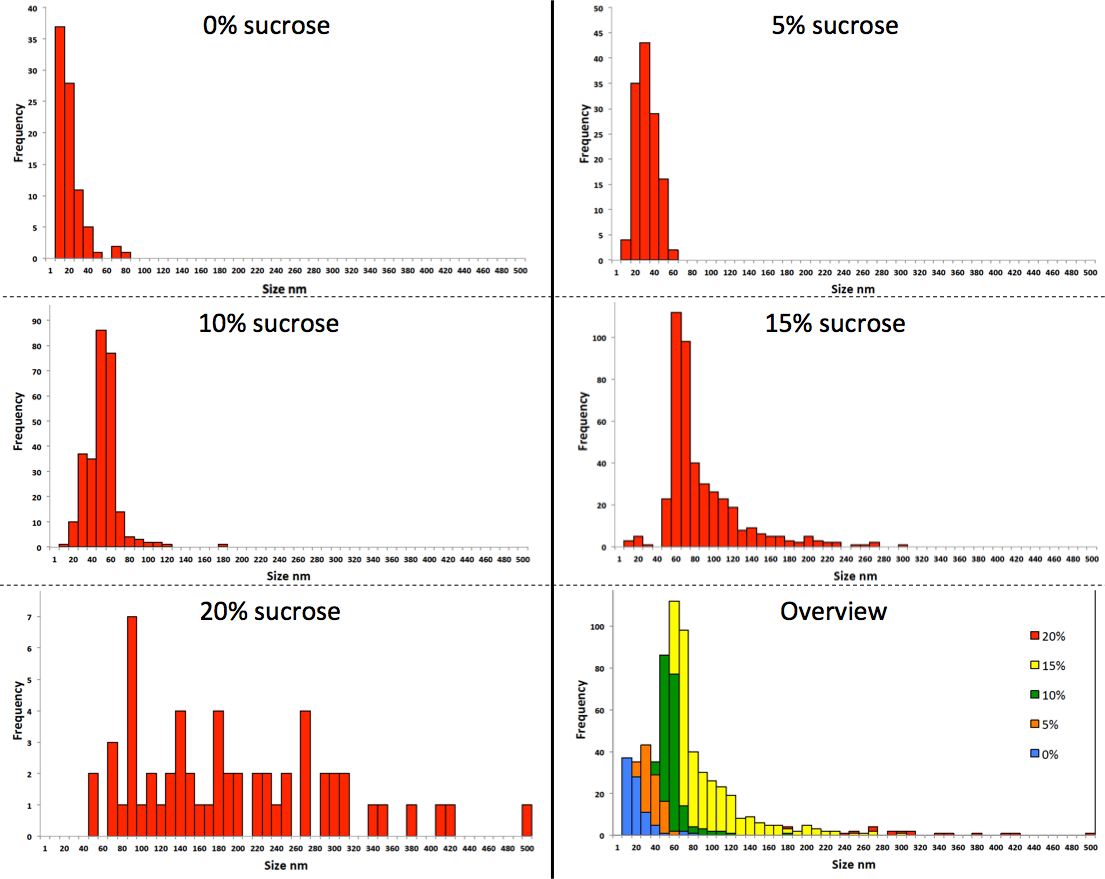

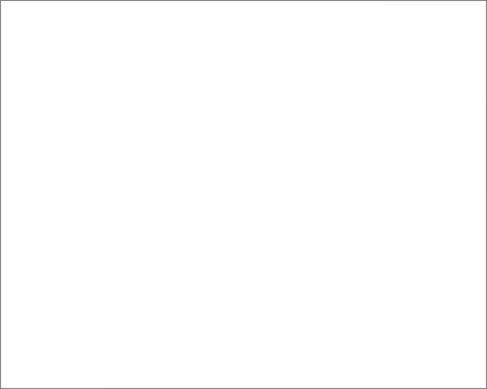


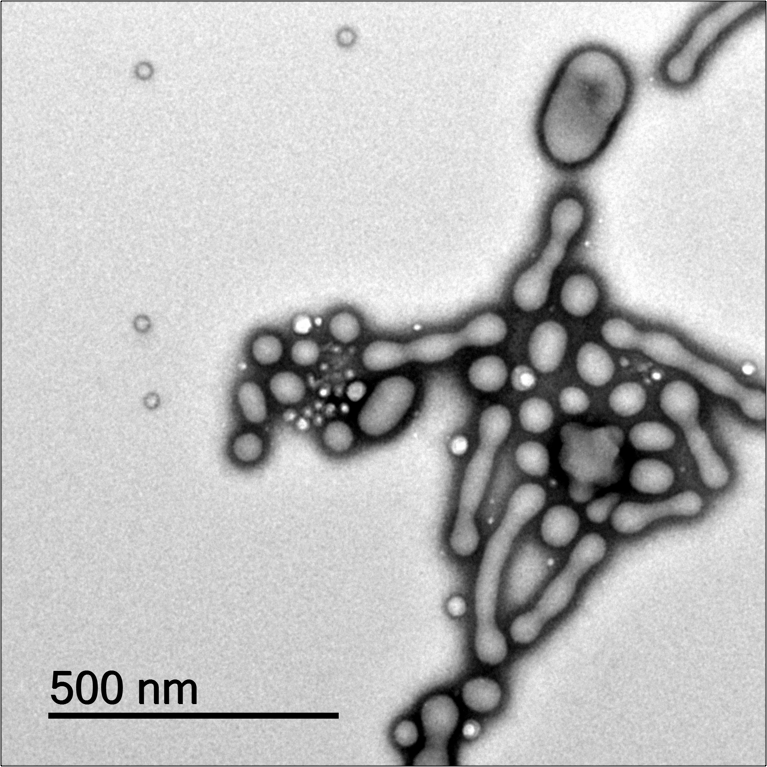
**Figure S5. Spherical and tubular polymersomes formed through film rehydration.** A TEM photomicrograph of PMPC-PDPA assemblies before separation by density gradient centrifugation.
